# Modular transitional nursing intervention improves pain-related self-management for cancer patients: Study protocol for a randomized controlled trial

**DOI:** 10.1097/MD.0000000000023867

**Published:** 2020-12-18

**Authors:** Beibei Miao, Yali Sun, Ling Gong, Wei Liu

**Affiliations:** School of nursing, Beihua University, Jilin, China.

**Keywords:** cancer, modular transitional nursing intervention, pain, protocol, self-management

## Abstract

**Objective:**

To explore the effect of modular transitional nursing intervention on the improvement of self-management of the patients with cancer pain.

**Method:**

This study will be conducted from March 2021 to May 2022 at Affiliated Hospital of Beihua University. The experiment was granted through the Research Ethics Committee of Affiliated Hospital of Beihua University (4348–019). Eighty patients are analyzed in our study. The patients will be included if they are between 18 and 70 years old and are diagnosed with cancer, the pain intensity score on moderate level, the pain lasts for more than 3 days, and the patients who have signed the written informed consent. While the patients will be excluded if they have a documented history of drug or alcohol abuse, and patients with limited performance, and patients have a surgery in the past 3 days. The primary result mainly expresses as intergroup differences in self-management disorders (Barriers Questionnaire-II) associated with the cancer pain. And the secondary results include the quality of life (QOL) and pain intensity. All the analyses are implemented with SPSS for Windows Version 20.0.

**Results:**

Table 1

**Table 1:** Outcome measures after modular transitional nursing intervention.

Variables	Nursing group (n = 40)	Control group (n = 40)	*P* value
Pain intensity (average)			
Pain intensity (maximum)			
Knowledge cancer pain			
Cognitive barriers			
Function			
Physical			
Role			
Emotional			
Cognitive			
Social			

will show the clinical outcomes between the 2 groups.

**Conclusion:**

A modular transitional nursing intervention appears to reduce pain in cancer patients.

**Trial registration number:**

researchregistry6262.

## 1 Introduction

Many cancer patients suffer from a variety of symptoms, which can affect their social and physical functioning.^[1,2]^ For the cancer patients, pain is one of the most burdensome and feared physical symptoms.^[3,4]^ A recent study indicated that in the cancer patients, the incidence of pain remains high: 64 percent in those with terminal, advanced and metastatic disease, and 59 percent in those receiving anti-cancer treatment.^[5]^ The nurses and physicians are involved in the management of daily cancer pain, but they often fail to detect and treat the syndromes of pain.^[6,7]^ The factors associated with the ineffective pain management can be divided into 3 categories, namely, patients, healthcare providers and healthcare system. In the healthcare system, “curing” is more concerned than “caring” for cancer patients, including the management of symptom. Health care providers often lack the knowledge and attention about the management of pain and patients are unwilling to report pain to physician. Fear of drug resistance, drug addiction and worry about the side effects can also affect their intake of painkillers. Despite pain education programs have increased the knowledge of nurse and patient in the management of pain, none of these programs, as far as we know, has improved the pain results.^[8–10]^ Goldberg et al^[11]^ have explored the educational effects of nurses on topics related to pain. Nevertheless, these interventions did not result in any obvious reduction in patients pain severity.

There is increasing evidence of the importance of transitional nursing interventions, nevertheless, the influence of educational interventions that utilize transition preparation for the patients with cancer pain during hospitalization has not been recognized. Hence, the purpose of our investigation is to assess the influence of Self Care Improvement through Oncology Nursing to reduce patients barriers and improve pain management in cancer patients.

## 2 Methods

This study will be conducted from March 2021 to May 2022 at Affiliated Hospital of Beihua University. The experiment was granted through the Research Ethics Committee of Affiliated Hospital of Beihua University (4348–019) and recorded in research registry (researchregistry6262). Sequence of random numbers is generated by a computer. Sequentially numbered sealed opaque envelopes are used for the concealment of random numbers. All the patients taking part in our experiment are randomly divided into control and study group and each group includes 40 patients.

### 2.1 Inclusion and exclusion criteria

The patients will be included if they are between 18 and 70 years old and are diagnosed with cancer, the pain intensity score on moderate level, the pain lasts for more than 3 days, and the patients who have signed the written informed consent. While the patients will be excluded if they have a documented history of drug or alcohol abuse, and patients with limited performance, and patients have a surgery in the past 3 days.

### 2.2 Intervention

In intervention group, Self Care Improvement through Oncology Nursing program is managed through the specially trained nurses. The counseling courses are divided into 3 modules. The first module, namely, pharmacologic management of pain, introduces a reliable pain evaluation, the use of the pain medications, and the communication about patient pain. The second module, namely the non-pharmacologic management of pain, contains the information about the influence of adjuvant pain managements and gives the patient a CD instructing them to independently perform the progressive muscle relaxation. While the third module (namely the discharge management related to pain) is designed to help the patients cope properly with potential self-management issues associated with pain in the process of transition to the outpatient care. Advice is provided on how to adhere to the self-management methods learned in the first and second modules after discharge. The checklist is provided to ensure appropriate discharge management is implemented. At the aim of ensuring that the consultation is tailored to the personal needs, but standardized in accordance with basic model, we offer an evaluation of the patients skills, knowledge, perceptions and attitudes of resources, and provide the indicative questions. Each question is associated with the intervention, for instance, offer the information on the types of application and the plans of pain medication. In control group, the patients are given care as usual, involving the standard pharmacological treatment of pain, but there is no standardized written material and teaching application, nor other evidence-based management options.

### 2.3 Outcomes

The primary result mainly expresses as intergroup differences in self-management disorders (Barriers Questionnaire-II)^[12]^ associated with the cancer pain. And the secondary results include the quality of life (QOL) and pain intensity.

### 2.4 Statistical analysis

Through utilizing the software of IBM SPSS Statistics for Windows, version 20.0, all the data can be analyzed (IBM Corp., Armonk, NY, USA). Afterwards, all the data are described with appropriate characteristics such as mean, median, standard deviation as well as percentage. The qualitative parameters for the groups are evaluated by *t* test. The categorical variables are determined by the χ^2^ tests. When *P* is less than .05, it is viewed to be significant in statistics.

## 3 Result

Table 1 will show the clinical outcomes between the 2 groups.

## 4 Discussion

Our experiment is the first to explore the effects of modular transitional nursing intervention to improve the management of pain and decrease patient barriers for the cancer patients. Pain remains one of the most painful and prevalent symptoms in the cancer patients, especially in the end-stage of this disease.^[13,14]^ Fourty percentage of patients with the early cancer or intermediate cancer and 90 percent of patients with the advanced cancer suffer from moderate to severe pain, and approximately 70 percent of cancer pain patients do not get enough pain relief.^[15]^ The pain associated with cancer is a main healthcare challenge.^[16]^ Despite the use of strong opioids may be appropriate for the survivors of moderate to severe pain, most of the cancer survivors do not need them for their pain problems. Furthermore, as more than 40 percent of the cancer survivors live more than 10 years, there is an increasing concern about the opioids long-term side effects and the risks of overdose, abuse, and misuse in the cancer survivors.^[17,18]^ Studies have indicated that the inadequate exchange of information between health care providers, the lack of communication with the patients and their family members in the transition period, and the high incidence of post-hospital adverse events suggest that the inadequate nursing transition from the hospital to family is frequent. Thus, we conduct this investigation to explore the effect of modular transitional nursing intervention on the improvement of self-management of the patients with cancer pain.

## 5 Conclusion

A modular transitional nursing intervention appears to reduce pain in cancer patients.

## Author contributions

Beibei Miao finishes the manuscript.

Ling Gong collectes data.

Wei Liu plans the study design.

Yali Sun reviews the study protocol

**Data curation:** Yali Sun.

**Formal analysis:** Yali Sun.

**Funding acquisition:** Wei Liu.

**Investigation:** Ling Gong.

**Methodology:** Ling Gong.

**Writing – original draft:** Beibei Miao.
